# Percutaneous Electrolysis for Patellar Tendinopathy: A Systematic Review and Meta-Analysis

**DOI:** 10.3390/life16050840

**Published:** 2026-05-19

**Authors:** Jorge Góngora-Rodríguez, Miguel Ángel Rosety-Rodríguez, Jorge R. Fernández-Santos, Carmen Ayala-Martínez, Pablo Góngora-Rodríguez, Manuel Rodríguez-Huguet

**Affiliations:** 1 Department of Nursing and Physiotherapy, University of Cádiz, 11009 Cádiz, Spain; jorge.gongora@uca.es (J.G.-R.); carmen.ayala@uca.es (C.A.-M.); manuel.rodriguez@uca.es (M.R.-H.); 2 Move-It Research Group, Biomedical Research and Innovation Institute of Cadiz, Puerta del Mar University Hospital, University of Cádiz, Plaza Fragela, s/n, 11003 Cádiz, Spain; miguelangel.rosety@uca.es; 3 Department of Physical Education, School of Education Science, University of Cádiz, 11519 Puerto Real, Spain; pablo.gongora@uca.es

**Keywords:** eccentric exercise, patellar tendinopathy, percutaneous electrolysis, physical therapy, rehabilitation

## Abstract

Patellar tendinopathy is a chronic musculoskeletal condition characterized by localized pain and functional impairment. This systematic review and exploratory meta-analysis aimed to synthesize current evidence on the effectiveness of percutaneous electrolysis (PE), combined with eccentric exercise, for improving functional performance in individuals with patellar tendinopathy. Following PRISMA guidelines and registration in PROSPERO (CRD420251233971), comprehensive searches were performed in Cochrane Library, PEDro, PubMed, ScienceDirect, Scopus, and Web of Science databases. Only two randomized controlled trials, published between 2016 and 2021, met the eligibility criteria and were included in the quantitative synthesis. Functional capacity recorded using the Victorian Institute of Sport Assessment-Patella (VISA-P) scale was the primary outcome. Both included studies presented some concerns regarding risk of bias. The pooled random-effects meta-analysis (REML estimation with Hartung–Knapp–Sidik–Jonkman adjustment) revealed no statistically significant difference favoring PE over control interventions (Hedges’ g = –0.10; 95% CI: –2.69 to 2.50; *p* = 0.72). Statistical heterogeneity was nominally low (I^2^ = 0%), although this metric is uninformative with only two studies. Between-group differences in both studies were below the minimal clinically important difference for the VISA-P scale. The certainty of evidence according to the GRADE framework was rated as very low. Given the extremely limited evidence base, these findings should be considered strictly exploratory. The very low certainty of evidence precludes definitive conclusions regarding the comparative effectiveness of PE. Larger, adequately powered randomized trials with standardized protocols and long-term follow-up are needed.

## 1. Introduction

Patellar tendinopathy, also known as Jumper’s Knee, is a painful syndrome localized in the anterior aspect of the knee associated with patellar tendon dysfunction. It is aggravated by increased physical demand, particularly in athletes engaged in repetitive jumping or running activities, and is typically reproduced by pressure over the area [1,2].

Generally, symptomatology manifests at the inferior pole of the patella, and following the current conceptual model of tendinopathies, it is characterized by tendon thickening associated with collagen degradation and neovascularization, consistent with current models of tendinopathy pathophysiology that describe a degenerative rather than purely inflammatory process [2,3,4]. This is primarily evident during daily activities such as descending stairs or sports activities that involve jumping. This process is increasingly understood as a consequence of cumulative tissue damage; in this context, fatigue failure models help illustrate how chronic strain drives the transition of soft tissues (such as ligaments and tendons) into pathological states [5]. Consequently, tendinopathies represent a major source of disability and training limitation in sports practice, with a prevalence estimated between 14% and 45% among professional and recreational athletes [1,6,7], often resulting in significant disability [8,9]. However, conventional treatments frequently fail to provide a complete resolution for this pathology [10,11]. Therefore, many patients experience persistent symptoms and limited long-term recovery.

Percutaneous Electrolysis (PE) is a minimally invasive physiotherapy technique consisting of the application of galvanic current directly to the injured tissue to achieve analgesic effects and induce an electrochemical reaction that reverses the tendon’s pathological cycle (this application stimulates a controlled local inflammatory response, aiming to promote collagen regeneration, modulate pain perception, and restore tendon homeostasis) [8,12,13,14]. The procedure must be ultrasound-guided to ensure precise application at the correct anatomical location, thereby facilitating collagen synthesis [15,16,17,18]. Typically, the intervention is complemented by an exercise protocol designed to stimulate tenocyte activation and achieve full functional recovery [6,9,19,20].

Clinical studies evaluating the efficacy of this technique highlight its benefits; however, these investigations present several limitations. Beyond being highly dependent on specialized equipment and the practitioner’s expertise and proficiency, many studies are characterized by small sample sizes, limited follow-up periods, and, in some cases, the absence of a control group for comparative analysis.

Although other reviews focusing on this invasive physiotherapy technique exist, these investigations do not focus exclusively on patellar tendinopathies. They often aggregate data from disparate anatomical sites with varying biomechanical profiles. This lack of specificity obscures the clinical efficacy of the technique in patellar tendinopathy. This is important due to the relevance of the patellar tendon in motor control and the biomechanical performance of the lower limb, enabling activities such as walking and jumping, as well as those associated with sports practice. Furthermore, previous syntheses have used heterogeneous outcome measures, failing to provide a clear consensus based on the VISA-P scale, the most sensitive and specific tool for this pathology. Consequently, a dedicated, quantitative synthesis is required to clarify whether the clinical adoption of PE in the patellar tendon is supported by robust functional data.

Given these limitations, a systematic synthesis of the available evidence is required to clarify whether the addition of PE to eccentric exercise may be associated with superior functional outcomes compared to control interventions. Therefore, the aim of this systematic review and exploratory meta-analysis was to evaluate the effectiveness of ultrasound-guided PE, combined with eccentric exercise, on functional improvement in individuals diagnosed with patellar tendinopathy, as measured by the VISA-P scale. Given the anticipated small number of eligible studies, this synthesis was designed as an exploratory, hypothesis-generating analysis rather than a definitive assessment of efficacy.

## 2. Materials and Methods

### 2.1. Review Protocol and Search Strategy

This systematic review and meta-analysis were conducted in accordance with the guidelines established in the Preferred Reporting Items for Systematic Reviews and Meta-Analyses (PRISMA 2020) statement. Consequently, the review was registered in the PROSPERO (International Prospective Register of Systematic Reviews) database, a dedicated tool used to ensure transparency, under the registration number: CRD420251233971.

The study question was defined using the PICOS framework. The study Population (P) included patients diagnosed with patellar tendinopathy confirmed by clinical and/or ultrasonographic findings. The Intervention (I) of interest was PE, alone or in combination with exercise-based rehabilitation, which was compared with any other therapeutic procedure: conservative physiotherapy, dry needling, sham needling, or other active or passive control interventions, with or without exercise, or absence of treatment (C). The primary Outcomes (O) assessed were the level of function or disability measured using the Victorian Institute of Sport Assessment–Patella (VISA-P) scale. Finally, the included Study Design (S) comprised Randomized Controlled Trials comparing PE treatment against a control group.

The search was conducted between October and November 2025 across international reference databases in Physiotherapy and Health Sciences, ensuring access to the largest possible number of articles: Cochrane Library, PEDro, PubMed, ScienceDirect, Scopus, and Web of Science. The latest search update was performed on 15 January 2026. Grey literature (OpenGrey) and trial registries (ClinicalTrials.gov) were searched, yielding no additional eligible RCTs. The search strategy combined Medical Subject Headings (MeSH) and free-text terms related to both the intervention and condition (Table 1). The main search term was ‘percutaneous electrolysis’ with the intention of correctly replicating the search across all databases and ensuring the inclusion of all articles related to treatment with the PE technique. No additional filters were applied during the searches, neither by time limitation nor by language. After removing duplicates, all identified records were subjected to a manual screening process conducted independently by two investigators. This selection process was performed in two stages: an initial screening of titles and abstracts, followed by a full-text review of potentially eligible studies. In both stages, any discrepancies were resolved through discussion or, when necessary, by a third reviewer. This filtering allowed for the selection of articles exclusively focused on the PE treatment of the patellar tendon. The decision to restrict the meta-analysis to studies utilizing the VISA-P scale was intentional. As the gold standard for assessing patellar tendinopathy, the VISA-P allowed pooling of clinically comparable outcomes. While this approach reduced the number of eligible studies, it ensured that the functional outcomes were clinically comparable and avoided introducing measurement heterogeneity into an already limited evidence base. This trade-off is acknowledged as a key limitation of the present synthesis.

### 2.2. Eligibility Criteria, Study Selection and Data Collection Process

To establish the final selection of studies, duplicate results were initially removed using the Rayyan web applicationaccessed on 15 January 2026. Subsequently, the screening process was conducted by reading the title and abstract, and finally, by full-text reading, following the pre-established selection criteria. The inclusion criteria were: Randomized Controlled Trials that included an intervention group with PE and a control group in the treatment of patellar tendinopathy, assessed using the VISA-P scale. Articles conducted on animal or cadaver models were excluded, as were studies with data previously reported in other investigations.

The inclusion criteria were intentionally designed to prioritize methodological homogeneity and internal validity. Quasi-randomized controlled trials (quasi-RCTs) were excluded to minimize the risk of bias associated with inadequate allocation concealment, following Cochrane recommendations for high-quality evidence synthesis. Similarly, studies utilizing generic scales or non-validated questionnaires were excluded in favor of the VISA-P scale. As the only functional tool specifically validated for patellar tendinopathy, the VISA-P ensures that the outcomes measured are clinically relevant to the pathology.

Data extraction for each selected article included the fundamental characteristics of each study, such as author, year of publication, total sample size and number of participants for each group, PE treatment parameters (number of sessions, current intensity, application time), follow-up duration, variables, and main results.

### 2.3. Quality Assessment of Studies and Risk of Bias

The risk of bias of each included study was assessed using Version 2 of the Cochrane risk-of-bias tool for randomized trials (RoB 2) [21] as the primary assessment instrument. This tool employs structured signaling questions across five domains: bias arising from the randomization process, deviations from intended interventions, missing outcome data, measurement of the outcome, and selection of the reported result. Independent domain-specific judgments (low risk, some concerns, high risk) were derived through the tool’s algorithm and summarized graphically.

Additionally, the Physiotherapy Evidence Database (PEDro) scale was applied as a complementary measure of overall methodological quality, given its widespread use in physiotherapy research [22]. The scale includes 11 items (the first not counted in the total score), yielding scores from 0 to 10, classified as poor (0–3), fair (4–5), good (6–8), or excellent (9–10). It is important to note that PEDro and RoB 2 assess overlapping but distinct constructs: PEDro provides a summary quality score, whereas RoB 2 evaluates domain-specific bias through a structured algorithm. Minor discrepancies between the two tools are expected and reflect methodological differences rather than inconsistencies in the review process.

### 2.4. Statistical Analysis

The standardized mean difference (SMD) was calculated using Hedges’ g to account for small sample size bias. Change scores (post-treatment minus baseline VISA-P) were used as the effect measure. Since standard deviations of change scores were not directly reported in either study, nor were pre-post correlation coefficients, standard deviations were estimated using the formula:

SD_change_ = ✓(SD_pre_^2^ + SD_post_^2^ − 2 × r × SD_pre_ × SD_post_)

where r represents the pre-post correlation coefficient. A conservative estimate of r = 0.5 was assumed for the primary analysis, consistent with recommended practice when pre-post correlations are not reported [23,24]. This value is considered conservative because, mathematically, lower assumed correlations yield larger estimated standard deviations of change and thus smaller standardized effect sizes with wider confidence intervals, reducing the risk of overestimating the treatment effect [23,24]. Sensitivity analyses were conducted across a range of correlation values (r = 0.3, 0.5, 0.7, and 0.9) to assess the robustness of findings to this assumption. For Abat et al. (2016) [25], data from the subgroup classified as “not healed” (final VISA-P < 90) were extracted for the primary analysis, as the baseline VISA-P values of this subgroup (52.5 ± 18.8 for control; 51.4 ± 17.9 for PE) were comparable to the López-Royo et al. (2021) [26] inclusion criterion (baseline VISA-P < 80). It should be noted that this stratification was based on the final treatment outcome rather than baseline severity. To assess the impact of this selection, a pre-planned sensitivity analysis was conducted using the complete Abat et al. (2016) [25] sample (n = 30 per group), with subgroup means and standard deviations recombined using standard formulae [24].

A random-effects model was employed using the restricted maximum likelihood (REML) estimator for between-study variance (τ^2^). Given the very small number of included studies (k = 2), the Hartung–Knapp–Sidik–Jonkman (HKSJ) adjustment [27] was applied to calculate confidence intervals for the pooled effect estimate, as recommended for meta-analyses with few studies. This adjustment provides more conservative confidence intervals and better controls the Type I error rate compared to the standard DerSimonian-Laird approach when k is small. Statistical heterogeneity was assessed using Cochran’s Q test and quantified using the I^2^ statistic, where I^2^ values of 25%, 50%, and 75% represent low, moderate, and high heterogeneity, respectively. However, interpretation of heterogeneity statistics was approached with caution given the limited number of studies, as these metrics have low power and precision with small k.

Formal assessment of publication bias (e.g., funnel plot asymmetry, Egger’s test) was not performed, as these methods require a minimum of approximately 10 studies to provide adequate statistical power [24]. The certainty of evidence for the primary outcome was assessed using the Grading of Recommendations, Assessment, Development, and Evaluations (GRADE) framework [28]. Results were additionally interpreted against the minimal clinically important difference (MCID) for the VISA-P scale, estimated at approximately 13 points [29].

This meta-analysis was conducted following the Preferred Reporting Items for Systematic Reviews and Meta-Analyses (PRISMA) guidelines and the Cochrane Handbook for Systematic Reviews of Interventions recommendations for meta-analyses with limited numbers of studies. All analyses were performed using R version 4.5.1 with the ‘meta’ (version 8.2-1) and ‘metafor’ (version 4.8-0) packages. Statistical significance was set at α = 0.05 (two-tailed).

## 3. Results

### 3.1. Study Selection

**Figure 1:**
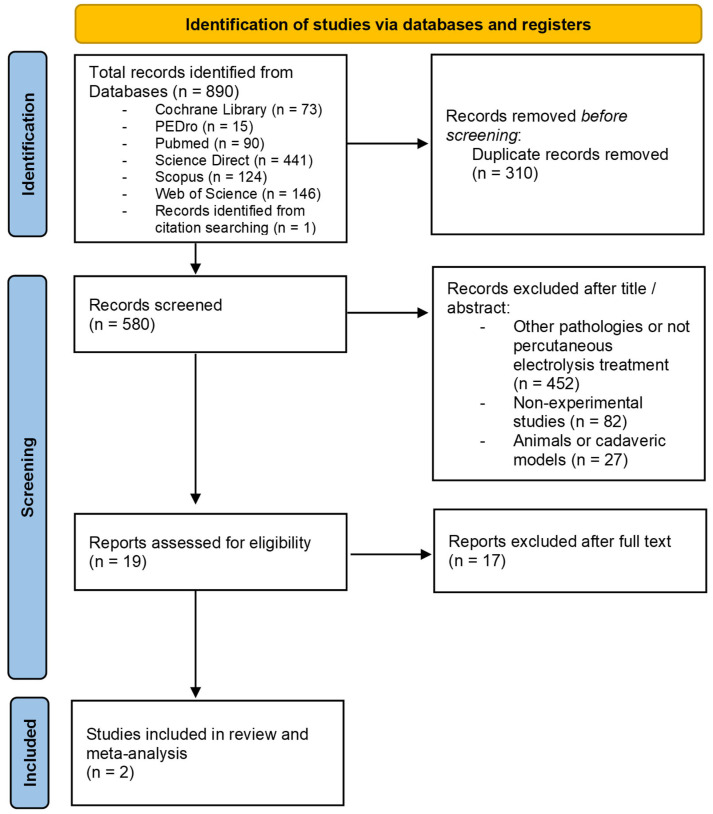
PRISMA flow diagram. Identification of the results obtained from the databases.

The process for selecting the articles included in this review and meta-analysis is presented in the PRISMA flow diagram (Figure 1). Ultimately, only two randomized controlled trials met all inclusion criteria and were retained for quantitative synthesis. Both trials specifically investigated PE in patellar tendinopathy and reported VISA-P outcomes, allowing for data pooling [25,26].

### 3.2. Sample Population Characteristics and Methodological Quality Assessment

The study by Abat et al. (2016) [25] involved a total of 60 participants who completed both the treatment and follow-up periods. Subjects were allocated into two groups of 30, receiving either PE treatment or conventional electrotherapy as a control. The sample was predominantly male. Selection criteria included an ultrasound-based diagnosis and the presence of symptoms for at least one month. Notably, participants receiving pharmacological treatment with fluoroquinolones were excluded, as these medications can alter tendon function.

For its part, the study by López-Royo et al. (2021) [26] included and followed 48 athletes presenting with pain at the inferior pole of the patella for at least three months and a VISA-P scale score below 80. Participants were allocated into three treatment groups (n = 16 per group), receiving either PE, dry needling, or sham needling, all combined with an eccentric exercise protocol. The sample consisted of 42 men and 6 women. Furthermore, patients treated with corticosteroids or anti-inflammatory drugs were excluded from the study.

**Figure 2:**
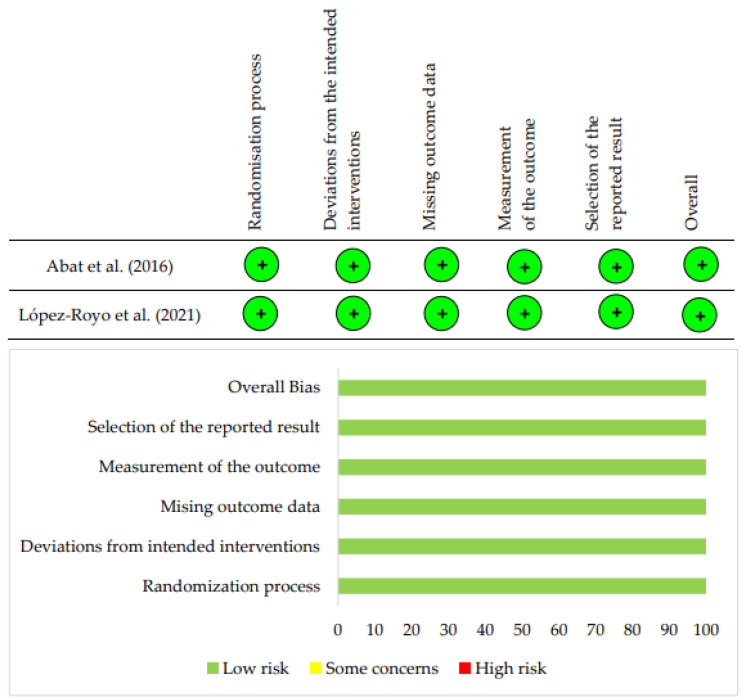
Risk of bias summary [25,26].

Regarding methodological quality, RoB 2 assessments indicated some concerns related mainly to allocation concealment and blinding rather than low overall risk of bias (Figure 2).

Furthermore, the scores obtained on the PEDro scale demonstrated fair [26] to good [25] quality, ensuring the consistency of the included articles while allocation concealment and blinding of subjects and therapists, which are inherent challenges in invasive physiotherapy interventions. These factors have been accounted for in the interpretation of the results (Table 2).

### 3.3. Outcomes Measurements and Assessment Time

The primary measure in the study by Abat et al. (2016) [25] was the VISA-P scale, utilized to assess symptom severity and the functional status of the patients; evaluations were conducted at baseline and at the conclusion of the 2-month treatment period. Within both groups, participants were categorized based on whether they reported scores above or below 90. Those with VISA-P scores exceeding 90 were considered recovered or asymptomatic; conversely, patients with lower scores were classified as symptomatic (this being the subgroup selected for inclusion in the meta-analysis). The ultrasonographic assessment for diagnosis focused on the bilateral comparison of tendon thickening, the presence of focal hypoechoic areas, tendon hypervascularity, and the presence or absence of intra-tendinous calcifications or cortical bone irregularities.

The study by López-Royo et al. (2021) [26] also focused its assessment on the VISA-P scale score as the primary outcome. Additionally, secondary outcomes included pain perception measured by the Visual Analogue Scale (VAS) and health-related quality of life via the Short Form-36 Health Survey (SF-36). The ultrasonographic characteristics of the patellar tendon were also analyzed following the same criteria as the previously mentioned study (thickening, neovascularization, focal hypoechoic areas, calcifications, or cortical irregularities). Follow-up evaluations were conducted at 10 and 22 weeks. For the meta-analysis, the group receiving PE was compared against the study’s control group.

### 3.4. Intervention Protocols and Effects of Treatments

The intervention protocol in the first of the analyzed studies [25] consisted of one ultrasound-guided PE session every two weeks over the two-month study period, totaling four sessions. In each session, galvanic current was applied at three anatomical sites: the superficial paratendon, deep paratendon, and the intratendinous area at the inferior pole of the patella, using an intensity of 2 mA. The control group received conventional electrotherapy (ultrasound, laser, and interferential currents) in 50-minute sessions, three times per week for two months. Furthermore, the treatment protocol in the study by Abat et al. (2016) [25] included eccentric exercise for both groups, specifically involving three sets of 15 repetitions of single-leg squats on a 25° incline, with 3-minute rest intervals between sets.

The protocol in the second study [26] included an eccentric exercise program common to all three groups, consisting of three sets of 15 single-leg squats on a decline board, performed twice daily. The invasive intervention involved needle insertion at three specific points, with a 3-second duration and an intensity of 3 mA for the application of PE. The control group received sham needling. Each group underwent four sessions, spaced two weeks apart over an eight-week treatment period.

For the invasive procedures in both studies, acupuncture needles measuring 0.25 × 25 mm were utilized [25,26]. The control groups in the selected trials exhibited distinct characteristics despite a shared baseline of eccentric exercise. This difference in control group design was accounted for in our qualitative analysis of the findings [25,26].

The results reported by Abat et al. (2016) [25] demonstrate that the combined therapy of PE and eccentric exercise may be more effective than conventional electrotherapy for patellar tendinopathy, yielding positive outcomes in functional assessment as measured by the VISA-P scale.

Focusing on the primary outcome measure, the study by López-Royo et al. (2021) [26] reported no superior benefits compared to the baseline treatment (the eccentric exercise protocol) in either the short- or medium-term follow-up. Specifically, no statistically significant differences were observed between treatment groups regarding the VISA-P scale. Most participants showed an increase in their VISA-P scores, achieving clinically relevant benefits regardless of the assigned treatment group. Furthermore, no statistically significant differences were found between groups for either mean or maximum pain scores recorded via the VAS, nor for health-related quality of life. Regarding the ultrasonographic assessment, a reduction in tendon thickening was observed across all groups.

### 3.5. Data Synthesis and Meta-Analysis

The individual study results are summarized in Table 3. Given the limited number of studies (k = 2), this meta-analysis should be considered exploratory, and the pooled estimates must be interpreted with extreme caution. The standard deviation of change scores was estimated using a conservative pre-post correlation coefficient of r = 0.5, as neither study reported this parameter [23]. To address the uncertainty associated with the small evidence base, the Hartung–Knapp–Sidik–Jonkman (HKSJ) adjustment was applied, which provides more conservative confidence intervals when the number of studies is small.

In the Abat et al. (2016) study [25], the PE group showed a mean VISA-P change of 11.9 points (SD = 16.4), while the control group improved by 9.4 points (SD = 16.8), yielding a small positive effect favoring PE (Hedges’ g = 0.15; 95% CI: −0.68 to 0.97). In the López-Royo et al. (2021) study [26], the PE group showed a mean VISA-P change of 16.9 points (SD = 14.6), while the control group improved by 20.4 points (SD = 10.4), yielding a small negative effect favoring the control group (Hedges’ g = −0.27; 95% CI: −0.97 to 0.43).

**Figure 3:**
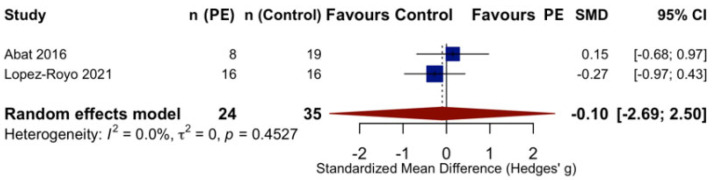
Random-effects meta-analysis [25,26].

The pooled effect estimates from the random-effects meta-analysis indicated no statistically significant difference between PE and control interventions for improving VISA-P scores in patients with patellar tendinopathy (Hedges’ g = −0.10; 95% CI: −2.69 to 2.50; *p* = 0.72) (Figure 3). The direction of the pooled effect was inconsistent across studies, with one study favoring PE and one favoring control.

Statistical heterogeneity was nominally low (I^2^ = 0%; τ^2^ = 0; Q = 0.56, df = 1, *p* = 0.45). However, the 95% confidence interval for I^2^ ranged from 0.0% to 99.8%, confirming that this metric is essentially uninformative with only two studies [30]. These heterogeneity estimates should not be interpreted as evidence of consistency between studies.

The between-group differences in VISA-P change scores were 2.5 points in Abat et al. (2016) [25] (favoring PE) and −3.5 points in López-Royo et al. (2021) [26] (favoring control). Both differences were substantially below the estimated MCID for the VISA-P scale of approximately 13 points [29], indicating that neither study demonstrated a clinically meaningful advantage of PE over control interventions. The pooled estimate was highly imprecise, with a confidence interval spanning from large effects favoring PE (Hedges’ g = 2.50) to large effects favoring control (Hedges’ g = −2.69), precluding any meaningful inference regarding comparative effectiveness.

The results remained consistent across all analytical variations (Table 4). Varying the assumed pre-post correlation from r = 0.3 to r = 0.9 did not alter the conclusion, although confidence intervals widened progressively (from [−2.28, 2.12] to [−5.80, 5.42]), reflecting the inherent instability of effect estimation with this limited evidence base. When 10-week data from López-Royo et al. (2021) [26] were replaced with 22-week follow-up data, the pooled estimate shifted slightly in the positive direction (Hedges’ g = 0.10; *p* = 0.20) but remained non-significant. Importantly, when the complete Abat et al. (2016) [25] sample was used instead of the VISA-P < 90 subgroup, the individual effect for Abat et al. (2016) [25] increased (Hedges’ g = 0.58), driven by the larger improvement observed in PE-treated patients who achieved VISA-P ≥ 90 at final assessment. However, the pooled estimate with López-Royo et al. (2021) [26] remained non-significant (Hedges’ g = 0.19; *p* = 0.73), and the between-group difference in the full sample (10.2 points) was still below the MCID of 13 points.

## 4. Discussion

This systematic review and exploratory meta-analysis found that the currently available randomized evidence does not demonstrate a statistically significant advantage of ultrasound-guided PE, when added to eccentric exercise, over control interventions for patellar tendinopathy (Hedges’ g = −0.10; 95% CI: −2.69 to 2.50; *p* = 0.72). The certainty of this evidence, assessed using the GRADE framework, was rated as very low, meaning that the true effect is likely to be substantially different from the estimate reported here. Accordingly, these findings should be understood as an early, hypothesis-generating synthesis that maps current evidence gaps rather than as proof of effectiveness or ineffectiveness of PE.

The between-group differences in VISA-P change scores (2.5 and −3.5 points) were well below the MCID of approximately 13 points, indicating that neither study demonstrated a clinically meaningful advantage of PE over control interventions. The direction of the pooled effect was inconsistent across studies, with Abat et al. (2016) [25] favoring PE (Hedges’ g = 0.15) and López-Royo et al. (2021) [26] favoring control (Hedges’ g = −0.27), further underscoring the uncertainty of the available evidence.

Therefore, this review should be understood primarily as an early, hypothesis-generating synthesis that maps the current evidence gaps rather than as definitive proof of effectiveness or ineffectiveness of PE. Nevertheless, several considerations warrant further discussion.

Primarily, the limited number of studies included in this analysis must be highlighted. This limitation stems from a selection process strictly restricted to trials where PE was the primary intervention for patellar tendinopathy compared against a control group receiving alternative treatments. Furthermore, inclusion was contingent upon the use of the VISA-P scale as an assessment tool to ensure that results were sufficiently comparable for data pooling within the meta-analysis.

Utilizing the VISA-P scale as a primary reference is an essential premise when assessing functional status in patellar tendinopathies. It is a reproducible and easily applicable tool that correlates symptomatology with functional capacity during various activities. Furthermore, it functions as a patient-reported outcome measure, ensuring that results depend on the patient rather than the evaluator, thus reducing observer bias. Its intuitive scoring system facilitates the monitoring of patient progression in both research settings and daily clinical practice [31,32,33]. The results reported by Abat et al. (2016) [25] were stratified by final VISA-P score, with patients classified as “not healed” (VISA-P < 90) or “healed” (VISA-P ≥ 90) at the end of treatment. For the primary meta-analysis, data from the “not healed” subgroup were selected due to clinical comparability of baseline values with the López-Royo et al. (2021) [26] sample. It should be acknowledged that this stratification was conditioned on the treatment outcome rather than on baseline severity, which may introduce selection bias. To address this concern, a sensitivity analysis using the complete Abat et al. (2016) [25] sample (n = 30 per group) was conducted, yielding a larger individual effect (Hedges’ g = 0.58) but a pooled estimate that remained non-significant (Hedges’ g = 0.19; *p* = 0.73), confirming that the primary conclusion is robust to this analytical decision.

An important source of clinical heterogeneity lies in the nature of the control interventions. Abat et al. (2016) [25] compared PE against conventional electrotherapy (ultrasound, laser, and interferential currents), which constitutes an active treatment comparison and addresses a superiority question. In contrast, López-Royo et al. (2021) [26] compared PE against sham needling, representing a placebo-controlled efficacy question. These are fundamentally different research questions, and their pooling necessarily assumes a broad common comparator (“PE + exercise vs. any alternative intervention”). With only two studies, stratified analysis or meta-regression is not feasible; however, the individual study effects are presented alongside the pooled estimate to allow readers to evaluate each comparison independently. This heterogeneity of control conditions is a key factor that may explain the inconsistent direction of effects between studies.

In contrast, the study by López-Royo et al. (2021) [26] suggested that the observed beneficial effects were primarily contingent upon physical exercise (specifically eccentric exercise) failing to find statistically significant differences favoring the use of PE. Furthermore, it is noteworthy that this research also emphasized the assessment of pain perception and quality of life as key secondary outcomes.

These findings are consistent with those reported in prior investigations focusing on the same pathology and utilizing the same assessment instrument. Such studies indicate that the application of PE in patellar tendinopathies may yield beneficial changes in VISA-P scores [20,34,35,36].

Although these studies did not meet the eligibility criteria for inclusion in the current meta-analysis, previous research supports the application of galvanic current to the patellar tendon, demonstrating positive effects on functional outcomes over both the short [34] and long term [20,26,35,37] The stratification of participants into groups based on their VISA-P scores is a consistent feature across these investigations [34,35,36], this approach allows for the comparison of effects within more homogeneous subgroups, it inherently limits blinding. Furthermore, despite reporting beneficial outcomes, the lack of a control group in these specific designs precludes a direct comparative analysis between different treatment modalities [35]. Similarly, the case report published by Muñoz-Fernández et al. (2021) [38,39] highlights positive outcomes regarding both functional recovery and pain relief, alongside significant ultrasonographic changes in tendon morphology.

Regarding structural outcomes, it is important to note that ultrasonographic changes often lag functional recovery. It is worth noting that observed changes may be limited when follow-up is conducted in the short term [25,26,31,32,34,36], as the biological processes involved in tendon tissue remodeling typically require a longer duration to manifest significant structural improvements. Furthermore, it is necessary to consider that ultrasonographic assessment is highly operator-dependent and, in many instances, is conducted in a qualitative manner, which may limit the objectivity of the structural findings [36,40]. Therefore, further research is warranted to elucidate the association between clinical symptomatology and structural adaptations, as these variables currently demonstrate a lack of significant statistical correlation [26,41]. Nevertheless, it is pertinent to specify that the successful application of the PE technique is strictly contingent upon the accurate identification of the lesion site. Furthermore, the therapeutic strategy (specifically whether a single-point approach or the delivery of galvanic current across multiple points is employed) must be considered, as detailed in the methodologies of the selected studies [25,26,31,32].

Accordingly, the observed findings align with results from prior investigations in which PE was utilized as a therapeutic modality. This consistency facilitates a deeper understanding of its underlying mechanism of action and its potential clinical utility in the management of this pathology [17,18]. PE induces pH alterations and a controlled inflammatory response that promotes collagen synthesis and tissue repair [16,17,42,43,44,45]. These findings could be crucial for elucidating the underlying mechanism of action of PE and clarifying its contribution to pathological resolution, especially when considering tendinopathies as a predominantly degradative process [2,4]. Beyond structural effects, PE modulates the autonomic nervous system and pain [13,14,39,46,47,48], while animal models suggest it facilitates extracellular matrix remodeling [40,41,49,50]. However, these physiological changes must be integrated into the multifactorial management of tendinopathy, where exercise remains the cornerstone of treatment [19,20,35,44,45,47,48,51,52,53].

On the other hand, a recurring challenge in PE research is the limited sample size included across studies. Despite this constraint, these clinical trials report significant benefits regarding pain perception and pressure pain thresholds [49,54]. Regarding follow-up duration, the outcomes derived from the combination of PE and eccentric exercise should be analyzed over longer periods, advocating for long-term exercise protocols [50] and extended longitudinal follow-up [20,22,35,37,43].

Focused on the PE treatment protocols, both studies [25,26,31,32] implemented comparable interventions. Nevertheless, it should be noted that further investigation is warranted to elucidate the musculoskeletal tissue adaptations and pain modulation mechanisms as a function of the applied current intensity [12,51,52,55]. Galvanic current intensity and application duration represent the primary parameters for regulating PE dosage. Most studies reach a consensus on utilizing short application periods—typically between 3 and 5 seconds—in conjunction with intensities ranging from 2 to 3 mA [10,11]. Furthermore, within the comparative framework against conventional electrotherapy, Abat et al. (2016) employs a distinct treatment frequency [25,31]. Regarding exercise regimens, the selected investigations utilize similar interventions [25,26,31,32]; however, it is necessary to highlight the importance of implementing phased progression and incorporating a greater variety of mechanical stimuli through therapeutic exercise [19,20,50,56]. This approach builds upon the well-documented potential of eccentric training to optimize functional outcomes [9,10].

Returning to the analysis of the results, it is pertinent to address the methodological quality of both studies, as reflected in their PEDro scale scores. The primary limitation identified was the challenge of participant blinding, a common hurdle in invasive interventions. Nevertheless, this methodological constraint can be mitigated through the implementation of sham needling protocols [26,32,49,53]. Consequently, this underscores the potential influence of the placebo effect on the observed clinical outcomes, a factor that must be carefully considered when interpreting the efficacy of invasive interventions [54,57]. Furthermore, an additional limitation lies in the difficulty of extrapolating these findings to both sexes due to the male-dominated nature of the study samples [26,32]. This disparity may be attributed to the specific demographic under investigation (athletes) where male sports have traditionally garnered greater research focus and visibility.

A notable strength of the analyzed studies is the comparison between two distinct invasive intervention modalities [26,32], this methodological approach aligns with investigations conducted in other anatomical regions where dry needling has also been compared against PE, both in conjunction with eccentric exercise programs [55,58], notwithstanding the divergent findings reported in these studies. Moreover, evidence supports that PE may provide superior patient tolerance compared to needle-based interventions performed without electrical current [49,59]. Consequently, it is estimated that PE yields more favorable clinical outcomes than dry needling in the management of painful symptomatology associated with musculoskeletal disorders [57,60]. Research in other anatomical regions, such as the shoulder [36,40,55,58,59,60,61,62,63], and elbow [61,62,63,64,65,66,67], has suggested positive outcomes for PE. However, these findings cannot be directly extrapolated to the patellar tendon due to its unique biomechanical environment and the high degree of uncertainty found in our specific meta-analysis. The discrepancy between the perceived clinical success of PE in various tendinopathies and the null findings in this study underscores the need for standardized, large-scale trials [56,65]. Furthermore, the cost-effectiveness of this therapy renders it as accessible as other invasive modalities or sham needling procedures [66].

Therefore, given the findings, there appears to be a consensus that exercise prescription (particularly eccentric exercise) constitutes the cornerstone of recovery in tendinopathy, being essential when combined with invasive techniques. In accordance with the initial objectives, the protocols implemented in the clinical trials included in this meta-analysis can be recommended. This is consistent with the findings reported in prior systematic reviews [10,11,56,67]. A critical finding of this study is the high level of uncertainty reflected in the pooled estimate’s lack of precision. The direction of effect was inconsistent between the two included trials, leading to a null overall effect. This inconsistency may stem from clinical heterogeneity in control groups and the small sample size, which effectively limits the power to detect a true difference if one exists.

The GRADE assessment rated the overall certainty of evidence as very low (Table 5), driven by serious concerns across all evaluable domains: risk of bias (lack of blinding), inconsistency (opposite effect directions), indirectness (heterogeneous control interventions), and very serious imprecision (confidence interval spanning large effects in both directions with a total sample of 59 participants). This rating indicates that the true effect of PE is likely to be substantially different from the point estimate and that future well-designed trials are very likely to change the current estimate. The present findings therefore cannot serve as a basis for clinical recommendations either for or against PE in patellar tendinopathy.

This review has several important limitations that must be considered when interpreting the findings. Overall, only two studies met the inclusion criteria, resulting in a total sample of 59 participants, which severely limits statistical power and the reliability of effect estimates. A meta-analysis with k = 2 cannot provide precise pooled estimates and should be considered exploratory. The control interventions differed substantially between studies (conventional electrotherapy vs. sham needling), representing different research questions and introducing clinical heterogeneity that cannot be resolved through statistical methods with so few studies. Follow-up durations were inconsistent (2 months in Abat et al. (2016) [25,31] vs. 10 or 22 weeks in López-Royo et al. (2021) [26], limiting the comparability of treatment effects across time.

The standard deviations of change scores were estimated using an assumed pre-post correlation of r = 0.5, as neither study reported this parameter. Although sensitivity analyses across r = 0.3 to 0.9 confirmed the robustness of the conclusion, the reliance on imputed values introduces additional uncertainty. The subgroup selected from Abat et al. (2016) [25] (VISA-P < 90) was stratified by final treatment outcome rather than baseline severity. While the baseline values were clinically comparable to the López-Royo et al. (2021) [26] sample, this outcome-conditioned selection introduces a potential risk of bias. A sensitivity analysis using the complete Abat et al. (2016) [25] sample confirmed that this decision did not alter the primary conclusion.

Formal assessment of publication bias was not feasible with only two studies. The geographic concentration of PE research in Spanish institutions and the overlap of authorship across the existing literature may increase the risk of selective publication of favorable results. The heterogeneity statistic I^2^ was nominally 0%, but its 95% confidence interval (0.0% to 99.8%) confirms that this metric has no interpretive value with k = 2. The original submission did not include a formal assessment of evidence certainty; the GRADE evaluation added in this revision (very low certainty) addresses this gap. Both studies had predominantly male samples, limiting generalizability across sexes. Finally, the focus on the VISA-P scale, while ensuring clinical comparability, excluded studies using other valid outcome measures that could have broadened the evidence base.

## 5. Conclusions

In conclusion, this exploratory meta-analysis found no statistically significant difference between PE combined with eccentric exercise and control interventions for patellar tendinopathy (Hedges’ g = −0.10; 95% CI: −2.69 to 2.50). The certainty of evidence, assessed using the GRADE framework, was very low, indicating that the true effect is likely to be substantially different from this estimate. The lack of statistically significant differences is primarily attributable to insufficient data rather than to demonstrated equivalence between interventions, and neither study demonstrated between-group differences exceeding the minimal clinically important difference for the VISA-P scale. While exercise-based interventions remain the cornerstone of tendinopathy management based on the broader literature, the specific added value of PE cannot be determined from the current evidence base. Future research should prioritize adequately powered, sham-controlled randomized trials with standardized PE protocols, long-term follow-up, and diverse populations.

## Figures and Tables

**Table 1:** Search strategy.

Database	Search String
Cochrane Library	(percutaneous electrolysis):ti,ab,kw OR (galvanic electrolysis):ti,ab,kw OR (EPI):ti,ab,kw OR (EPTE):ti,ab,kw AND (patellar tendinopathy):ti,ab,kw OR (jumper’s knee):ti,ab,kw
PEDro	Abstract & Title: percutaneous electrolysis AND patellar tendinopathy
Pubmed	(“Electrolysis”[MeSH] OR “percutaneous electrolysis”[Title/Abstract] OR “galvanic electrolysis”[Title/Abstract] OR “EPI”[Title/Abstract] OR “EPTE”[Title/Abstract]) AND (“Patellar Tendinopathy”[MeSH] OR “patellar tendinopathy”[Title/Abstract] OR “jumper’s knee”[Title/Abstract] OR “patellar tendon”[Title/Abstract])
ScienceDirect	Title, abstract, keywords: (“percutaneous electrolysis” OR “EPI” OR “EPTE”) AND (“patellar tendinopathy” OR “jumper’s knee”)
Scopus	TITLE-ABS-KEY (“percutaneous electrolysis” OR “galvanic electrolysis” OR “EPI” OR “EPTE”) AND TITLE-ABS-KEY (“patellar tendinopathy” OR “jumper’s knee” OR “patellar tendon”)
Web of science	TS = (“percutaneous electrolysis” OR “galvanic electrolysis” OR “EPI” OR “EPTE”) AND TS = (“patellar tendinopathy” OR “jumper’s knee” OR “patellar tendon”)

**Table 2:** PEDro Score.

Author (Year)	PEDro Scale Score Details
Abat et al. (2016) [25]	7/10 [Eligibility criteria: Yes; Random allocation: Yes; Concealed allocation: Yes; Baseline comparability: Yes; Blind subjects: No; Blind therapists: No; Blind assessors: Yes; Adequate follow-up: Yes; Intention-to-treat analysis: No; Between-group comparisons: Yes; Point estimates and variability: Yes.]
López-Royo et al. (2021) [26]	5/10 [Eligibility criteria: Yes; Random allocation: Yes; Concealed allocation: No; Baseline comparability: No; Blind subjects: No; Blind therapists: No; Blind assessors: Yes; Adequate follow-up: Yes; Intention-to-treat analysis: Yes; Between-group comparisons: No; Point estimates and variability: Yes.]

Eligibility criteria item does not contribute to total score. Scores have been confirmed on PEDro website.

**Table 3:** Summary of individual study results and meta-analysis.

Study	n (Intervention)	n (Control)	Intervention VISA-P Change Mean (SD)	Control VISA-P Change Mean (SD)	Hedges’ g	95% CI
Abat et al. (2016) [25]	8	19	11.9 (16.4)	9.4 (16.8)	0.15	−0.68 to 0.97
López-Royo et al. [26](2021)	16	16	16.9 (14.6)	20.4 (10.4)	−0.27	−0.97 to 0.43
Pooled estimate (random-effects)	24	35	--	--	−0.10	−2.69 to 2.50 (*p* = 0.72)

Notes: VISA-P change represents post-treatment score minus baseline score (higher values indicate improvement). Standard deviations of change estimated assuming pre-post correlation r = 0.5. Pooled estimate calculated using random-effects model with REML estimator and Hartung–Knapp adjustment. Heterogeneity: I^2^ = 0%, τ^2^ = 0, Q = 0.56 (*p* = 0.45). Sample sizes for Abat et al. (2016) [25] correspond to the VISA-P < 90 subgroup at final assessment (PE: n = 8; Control: n = 19), selected for clinical comparability with the López-Royo et al. (2021) [26] baseline inclusion criterion (VISA-P < 80).

**Table 4:** Sensitivity analyses: effect of analytical decisions on pooled estimates.

Analysis	Pooled *SMD*	95% CI	*p*-Value	τ^2^	I^2^	Q	*p*(Q)
Primary (subgroup, 10 wk, r = 0.5)	−0.10	−2.69 to 2.50	0.72	0.00	0%	0.56	0.45
Sensitivity: r = 0.3	−0.08	−2.28 to 2.12	0.72	0.00	0%	0.41	0.52
Sensitivity: r = 0.7	−0.13	−3.45 to 3.19	0.71	0.00	0%	0.92	0.34
Sensitivity: r = 0.9	−0.19	−5.80 to 5.42	0.74	0.24	0.6%	2.52	0.11
22-week follow-up (subgroup)	0.10	−0.34 to 0.54	0.20	0.00	0%	-	-
Full sample Abat et al. [25](10 wk)	0.19	−5.16 to 5.54	0.73	0.26	0.7%	-	-
Full sample Abat et al. [25] + 22 wk	0.38	−2.72 to 3.48	0.36	0.03	0.2%	-	-

Notes: SMD = standardized mean difference (Hedges’ *g*); CI = confidence interval; r = assumed pre-post correlation for estimating SD of change. All models used the random-effects approach with REML estimator and Hartung–Knapp–Sidik–Jonkman adjustment. The primary analysis is highlighted in bold. “Full sample” analyses used recombined data from both VISA-P subgroups (<90 and ≥90 at final assessment) in Abat et al. (2016) [25]. Q-statistic and its *p*-value are reported only for analyses where both Abat subgroups contribute to pooling. The non-significant pooled result is consistent across all seven analytical variations, with all between-group differences remaining below the minimal clinically important difference of 13 VISA-P points. Full sample analyses used recombined data from both VISA-P subgroups (<90 and ≥90 at final assessment) in Abat et al. (2016) [25], yielding n = 30 per group. Subgroup means and standard deviations were recombined using standard formulae [24].

**Table 5:** GRADE Summary of Findings: PE + eccentric exercise vs. control for patellar tendinopathy.

GRADE Domain	Assessment	Rationale
Outcome	VISA-P change score	Post-treatment minus baseline VISA-P score
No. of studies (participants)	2 RCTs (n = 59)	PE = 24; Control = 35
Effect estimate	Hedges’ g = −0.10	95% CI: −2.69 to 2.50
Risk of bias	Serious (−1)	Some concerns in both studies (RoB 2); lack of participant and therapist blinding in invasive procedures
Inconsistency	Serious (−1)	Direction of effect opposite between studies (Abat et al.: g = +0.15; López-Royo et al.: g = −0.27)
Indirectness	Serious (−1)	Control interventions differed substantially (active electrotherapy vs. sham needling), representing different research questions
Imprecision	Very serious (−2)	Extremely wide confidence interval spanning large effects in both directions; total sample size well below optimal information size
Publication bias	Not assessable	Formal assessment not possible with k = 2; geographic concentration of research and authorship overlap noted
Overall certainty of evidence	⊕◯◯◯ VERY LOW	Starting from HIGH (RCTs), downgraded by 5 levels (capped at VERY LOW). The evidence does not permit reliable conclusions about the comparative effectiveness of PE.

Notes: GRADE = Grading of Recommendations, Assessment, Development, and Evaluations; RCT = randomized controlled trial; PE = percutaneous electrolysis; VISA-P = Victorian Institute of Sport Assessment–Patella; RoB 2 = Cochrane Risk of Bias tool version 2. Certainty of evidence starts at HIGH for RCTs and is downgraded by one level for each serious concern (or two for very serious). The symbol ⊕◯◯◯ indicates very low certainty.

## Data Availability

Data are contained within the article.

## Abbreviations

The following abbreviations are used in this manuscript:

PE	Percutaneous Electrolysis
VAS	Visual Analogue Scale
VISA-P	Victorian Institute of Sport Assessment-Patella
